# Genital examination training: assessing the effectiveness of an integrated female and male teaching programme

**DOI:** 10.1186/s12909-016-0822-y

**Published:** 2016-11-22

**Authors:** Lynn McBain, Susan Pullon, Sue Garrett, Kath Hoare

**Affiliations:** 1 Department of Primary Health Care & General Practice, University of Otago, PO Box 7343, Wellington, Wellington South 6242 New Zealand; 2 Education Unit, Dean’s Department, University of Otago, Wellington, New Zealand

**Keywords:** Medical student, Intimate physical examination, Genital teaching associate, Simulation training, Teaching techniques

## Abstract

**Background:**

Learning to undertake intimate female and male examinations is an important part of medical student training but opportunities to participate in practical, supervised learning in a safe environment can be limited. A collaborative, integrated training programme to provide such learning was developed by two university teaching departments and a specialist sexual health service, utilising teaching associates trained for intimate examinations in a simulated clinical educational setting. The objective of this research was to determine changes in senior medical students’ self- reported experience and confidence in performing male and female genital examinations, before and after participating in a new clinical teaching programme.

**Methods:**

A quasi-experimental mixed methods design, using pre and post programme questionnaires and focus groups, was used to assess the effectiveness of the programme.

**Results:**

The students reported greatly improved skill, confidence and comfort levels for both male and female genital examination following the teaching programme. Skill, confidence and comfort regarding male examinations were rated particularly low on the pre-teaching programme self- assessment, but post-programme was rated at similar levels to the female examination.

**Conclusions:**

This integrated female–male teaching programme (utilising trained teaching associates as simulated patients in a supervised clinical teaching environment) was successful in increasing senior medical students’ skills and levels of confidence in performing genital examinations. There were differences between female and male medical students in their learning. Suggestions for improvement included providing more detailed instruction to some clinical supervisors about their facilitation role in the session.

**Electronic supplementary material:**

The online version of this article (doi:10.1186/s12909-016-0822-y) contains supplementary material, which is available to authorized users.

## Background

Medical students find learning to do intimate clinical examinations challenging [1, 2]. Due to the sensitive nature of these examinations for patients as well as the reticence of some students, opportunities to gain skills in this area may not be readily available in the course of current medical school teaching programmes [3, 4] Opportunistic learning of first-time intimate examination skills only in clinical settings has long been identified as an inconsistent and ineffective method of ensuring that all students gain the necessary skills in ways that are respectful of patients and feasible for busy clinical staff [5].

With changing student demographics, including increased numbers of female medical students, and increasingly diverse ethnicities and cultural norms, [6] new ways of teaching in this area (often labour and/or resource intensive) need investigation and thorough evaluation. New teaching methods are continuously being explored to decrease student anxiety levels and to provide a safe environment for students to improve performance prior to practicing these examinations on patients in clinical settings. The use of audio visual material, mannequins, [7] tutorials and teaching associates [8–11] are common teaching modalities employed to improve confidence and competence. Internationally there has been a shift towards the use of paid, trained gynaecological teaching associates (GTAs) especially for learning to examine the female pelvis [12]. However there have been few studies investigating the effectiveness of female and male genital examination training using Teaching Associates (TAs) undertaken as part of the same programme [8, 13].

Some criteria for evaluating simulation learning programmes have been developed. A supportive learner-centred milieu, with access to expert tutors, and the subsequent ability for any programme to ‘map onto clinical experience’ are considered important features to demonstrate [14].

The combined use of teaching modalities has been shown to be effective. For example, the addition of a standardised patient to a simulation model and use of electronic feedback [13], the use of standardised patients to teach genital examination prior to using the mechanical simulation, [15] and the use of an online learning module viewed immediately prior to a simulated class session [16].

With increasing numbers of medical students in successive classes and student feedback suggesting a learning/teaching deficit, a need was identified at the University of Otago, Wellington, New Zealand to improve first-time genital examination teaching at an appropriate time in the curriculum for all students, especially those inclined to be reticent about such examination practice.

Strategies for teaching genital examination previously included opportunistic teaching in various clinical settings, the use of mannequins (female examination only), and scheduled teaching of speculum examination and smear taking in a family planning clinic. There was no programmed male genital examination teaching.

In 2012 a group of clinical teachers and academics drawn from the Departments of Primary Health Care and General Practice (PHC&GP), Obstetrics and Gynaecology (O&G) and the Wellington Sexual Health Service (WSHS) met with the aim of developing a teaching intervention (a new programme component) that would meet the objectives of ensuring that every student had an opportunity to undertake both a first-time female and male genital examination in a safe and learner-supportive environment. The WSHS is a direct-access specialist service based in the community, staffed by sexual health physicians and clinical nurse specialists. The programme was first implemented in 2013.

This programme component (henceforth referred to as ‘the programme’) was deliberately positioned as a springboard for further learning in clinical workplaces, especially in upcoming clinically-based, general practice and obstetrics and gynaecology rotations - part of the Advanced Learning in Medicine phase (Years 4–6) of the medical degree curriculum.

The aim of this study was to determine changes in senior medical students’ self- reported experience and confidence in performing male and female genital examinations, before and after undertaking the new programme. This paper reports on the design of the teaching programme and the pre and post questionnaire evaluation results.

## Methods

### Population

The study population included all 84 medical students in year five (of a six year undergraduate degree programme) at the University of Otago, Wellington in 2013.

### The programme

**Fig. 1:**
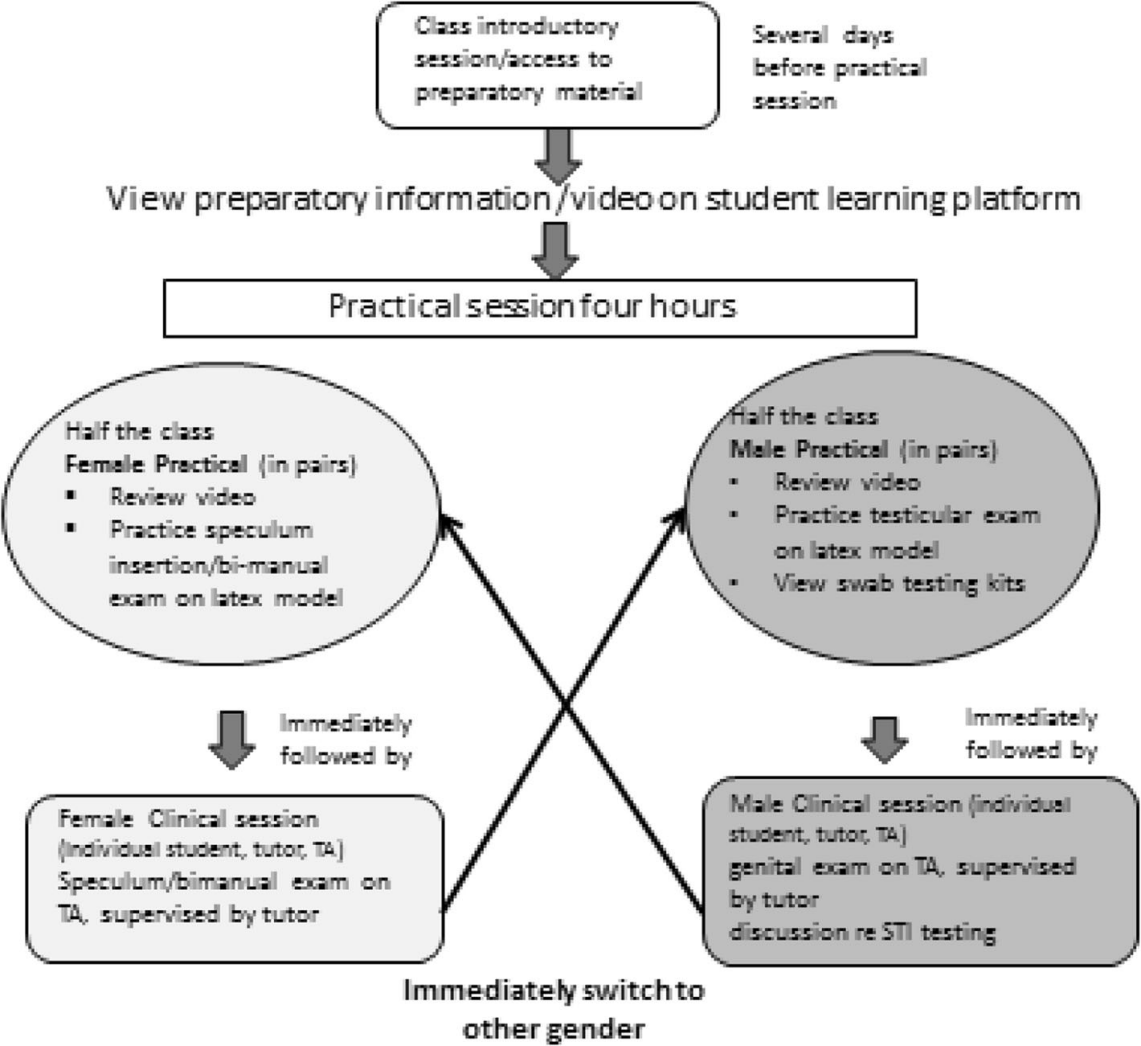
Student learning pathway for male/female genital exam

The genital examination teaching programme consists of an introductory session followed by a practical session several days later for all 5^th^ year medical students. The programme ensures that all students successfully undertake both a female and male examination. The learning takes place as part of one of several General Practice modules within the six year medical degree. See Fig. 1 for a visual depiction of the programme.

The introductory session outlines the planned programme, and enables students to ask questions and air any concerns. Preparatory material is available about genital examination processes and procedures on a secure online learning platform, including appropriate video-recordings. Students are expected to familiarise themselves with these resources before the day of the practical session.

Each 3–4 h practical session can accommodate up to 16 medical students at a time by running four parallel streams of dedicated clinic rooms (two for male, two for female examination), each using Teaching Associates (TAs) and clinical tutors. Half the student group undertake the female-specific preparatory learning activities and the female clinical examination first, then follow with the male-specific preparatory learning activities and male examination. The other half group undertake their learning in reverse order.

The organisation of 16 students, up to eight TAs, four clinical tutors, and TA and tutor reserves in any one practical session is complex but runs smoothly with efficient administration. During the teaching sessions the program convenor and administrator are available to circulate, assist with logistics and discuss and answer any questions the students may have.

Male and female TAs are recruited and trained by WSHS. The TA role is to be a real life patient so students can practice a genital (not breast or rectum) examination, and to provide patient feedback. Recruitment is initiated via posters placed in the waiting room of WSHS and/or word of mouth. Following an expression of interest, potential TAs are invited to a one-hour training session run by experienced WSHS staff. Following the session attendees are asked to indicate if they still want to be involved. Those who want to participate are then rostered to each of the practical sessions. TAs are paid for their services at the same rate as other simulated patients.

Experienced clinical teachers are recruited from the Departments of PHC&GP (vocationally trained general practitioners), O&G (consultants, senior registrars), and the WSHS (sexual health physicians, clinical nurses specialists). Tutors from all disciplines initially met together and agreed on the intended learning outcomes and the format for the sessions.

On the day of the practical session, students first prepare by working together in pairs to re-review a video recording of the examination they are about to undertake, and in turn practice the examination procedure on fabricated models. Prior to the female examination, students practice speculum insertion and bimanual examination. Prior to male examination, students practice testicular examination and view swab testing kits.

Individual students then immediately proceed to an appropriately equipped clinical examination room with a trained TA and a clinical tutor. Each student undertakes the examination, talking appropriately to the TA and guided throughout by the clinical tutor. During the female examination, the student is expected to correctly use a speculum to visualise the cervix, and undertake a bimanual examination. During the male examination, students undertake examination of the genital area, including testicular examination. Feedback, and testing for sexually transmitted infections is discussed but swabs are not taken. Constructive feedback is given and discussed by both the tutor and TA. Staff are available to assist students with debrief if required, although this has rarely proved necessary.

### Programme evaluation

The evaluation was undertaken using a mixed methods approach. Students were asked to complete pre and post teaching programme questionnaires. Following the programme successive groups of students were also invited to participate in focus groups. Focus groups were also undertaken with two cohorts of students in their final clinical year (year 6), one cohort who had not been part of the programme and a subsequent cohort who had participated in the programme. Focus group results will be reported separately.

Each student in the cohort was asked to complete pre and post programme questionnaires. Students were asked to rate items as “Non-existent”, “Poor”, “Adequate”, “Good”, or “Excellent”. They then rated their *skill level*, *confidence* and *comfort* with regards to completing both male and female exams, again using “non-existent”, “poor”, “adequate”, “good”, or “excellent”. Finally there was a free text field where students could include comments on both the pre and post questionnaires. The post programme questionnaire differed slightly in that questions six, seven and eight were asked about the current teaching intervention, rather than past experience (See Table 1). The questionnaires are also available as Additional files 1 and 2.

**Table 1:** Standardised student evaluation form questions

Pre-teaching Programme Questions
1. The adequacy of education to support your learning about genital examination?
2. The adequacy of educational materials (i.e. books, videos, access to internet and so forth) to support your learning about genital examination
3. The quality of dedicated instruction you have been given in relation to genital examination
4. The level of preparedness to complete genital examinations
5. The opportunities to observe genital examinations to date
6. The opportunities to participate in genital examinations to date
7. The supervision by clinicians involved in genital examination whilst in previous clinical practice runs
8. The level of feedback received on your genital examinations to date
9. Your skill level with genital examination^a^
10. Your confidence to complete genital examinations^a^
11. Your comfort with completing genital examinations^a^
Please include any additional comments relating to your previous learning and experience with genital examinations^b^

^a^Asked to rate male and female examinations separately

^b^Free text

Questionnaires were linked by a unique, non-identifiable number. Students were asked to use the last six digits of their mobile phone number. Students were given the option of omitting the code on the post questionnaire if they objected to their pre and post data being matched.

### Analysis

For ease of descriptive statistical calculation, the questionnaire responses were assigned numerical values as follows: non-existent = 1, poor = 2, adequate = 3, good = 4, excellent = 5. Responses were entered into an access database and analysed using SAS enterprise Guide V4.3. For linkable questionnaires Wilcoxon signed-rank tests were used to compare pre and post scores for all questions and *P* values were reported. We also analysed scores by student gender. The difference in overall means for Questions 1 to 8 by student gender were analysed using a Wilcoxon rank-sum test. Differences in how students rated their pre programme skill level for male and female examinations were analysed using Wilcoxon signed-rank tests. The difference between pre and post responses for Questions 9 through to 11 were also analysed by the gender of the student. A Wilcoxon rank-sum test was performed on the difference in test scores. Free text comments were reviewed by 3 investigators and grouped into obvious themes.

Ethical approval for this research was granted by the University of Otago Ethics Committee (ref:D13/120).

## Results

Of the 5th year student cohort for 2013, 81/84 students (96.4%) completed the pre-programme questionnaire and 80/84 students (95.2%) completed the post programme questionnaire. Of the cohort, 62/84 (74%) pre and post questionnaires were able to be matched using the unique identifier. Questionnaires were unable to be matched if participants had either declined consent by not submitting a code or had illegible codes. Twenty eight (45%) of the matched respondents were male and 34 (55%) female.

**Fig. 2:**
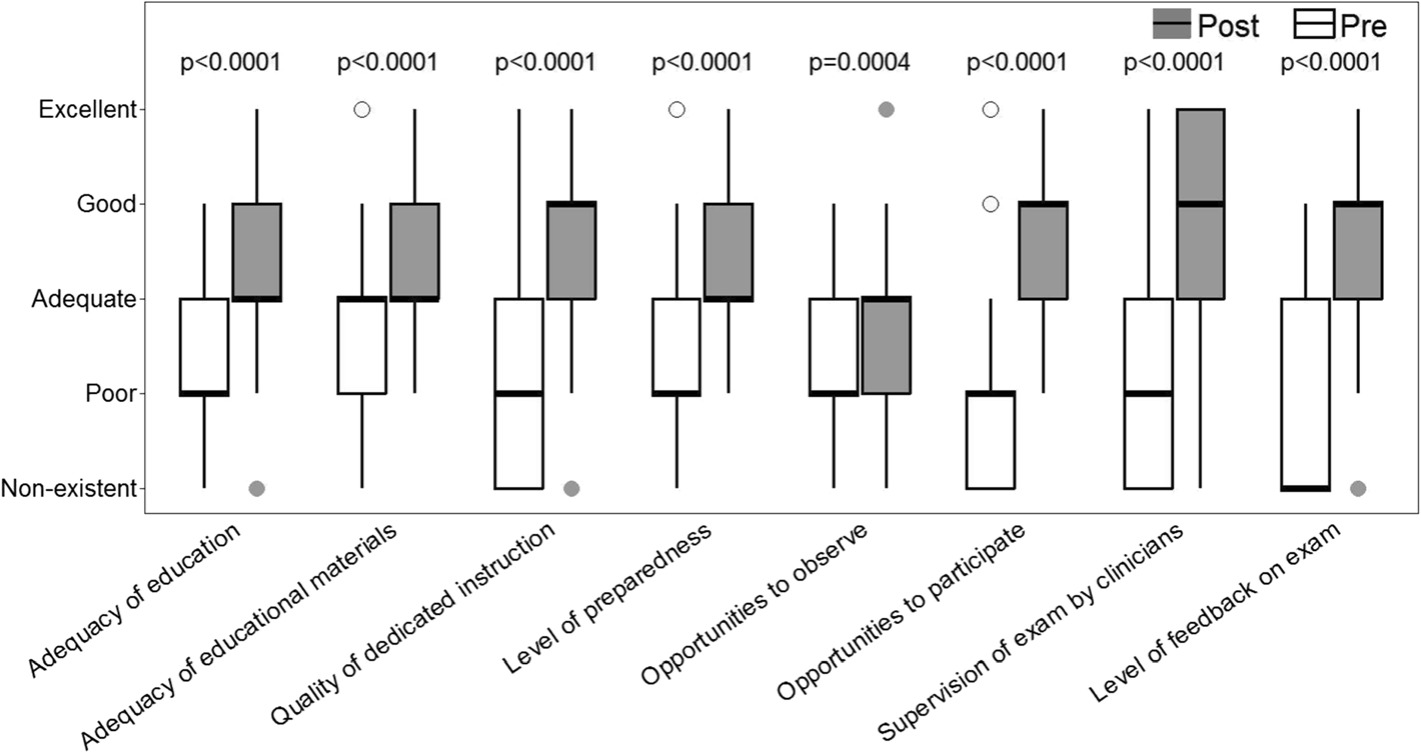
Student Self-Assessed pre and post genital exam teaching programme box plot medians (*thick line*), interquartile ranges, lines to most extreme point with 1.5 of the interquartile range and dots for extreme values (with *p* value) medians, and interquartile ranges (with *p* value)

When comparing the pre-teaching programme questionnaire results with the post-teaching programme, students’ self-assessments showed significant improvement after the teaching programme (Fig. 2).

Table 2 shows the students’ self-rated skill, confidence and comfort level for male and female genital exams pre and post teaching intervention. Students considered themselves less skilful, confident and comfortable at performing male genital exams compared to female exams prior to the teaching intervention. Following the teaching programme skill, confidence and comfort had improved for both female and male exams, but improved more for male exams.

**Table 2:** Students self-rated skill, confidence and comfort level (for male and female genital examination) pre and post programme

		Female genital exam			Male genital exam		
		Median	Interquartile range	*P* value	Median	Interquartile range	*P* value
Skill level	Pre	2	1–3		1	1–2	
	Post	3	3–4	<.0001	3	3–4	<.0001
Confidence level	Pre	2	1.5–3		2	1–2	
	Post	3	3–4	<.0001	3	3–4	<.0001
Comfort level	Pre	2	2–3		2	2–3	
	Post	3	3–4	<.0001	4	3–4	<.0001

Completion of a simple gender analysis identified there were differences between male and female students. For questions one to eight the median overall male student self-assessment score was slightly higher than the female students (median 2, interquartile range 2–3, versus 2, 1.1–3, on pre-intervention; 3.8, 3–4, versus 3.8, 3–4 on post-intervention), however these were not significant (*p* = 0.19 and 0.93). For the questions assessing skill, comfort and confidence with male and female examinations, both genders rated their pre-programme skill level for female examination much higher than for male examination, however only the results for female students were significant (Male students *p* = 0.14; Female students *p* = <0.0001). We also examined changes from the pre to post score means according to student gender, but none of the differences in student gender were significant.

Following the numbered responses in the questionnaire, students were invited to comment on their “*previous learning and experience with genital examinations”* (pre) and their “*learning and experience with genital examinations in this Year 5 GP module”* (post). The grouped themes (illustrated with quotes) from these comments are outlined in Table 3. Of the 62 linked questionnaires, 22 students included comments in the pre questionnaire and 33 in the post questionnaire. The pre teaching themes were largely based around lack of opportunity to participate in genital examinations on real patients, particularly male. The post teaching comments were largely positive, with a few helpful suggestions for improvement.

**Table 3:** Themes from free text field of questionnaire

Pre teaching programme themes	Post teaching programme themes
Lack of exposure to male examination ▪ no opportunities to examine males during O&G module ▪ Male genital examination - > non-existent ▪ O&G was adequate for female genital exam preparations, however we have had no exposure to male examinations ▪ Have not had any structured teaching in male genital examination ▪ Haven’t really done any teaching/learning on males ▪ More or less non-existent teaching or experience on genital examination to date Opportunistic exposure only ▪ Some minimal incidental exposure in clinical practice ▪ Only have female experience due to [undertaking a] summer studentship ▪ Previous experience with female genital examination was observing during pap smear on two General Practice occasions ▪ Observed one or two in 4th year Limited or no exposure ▪ A topic that has not been addressed previously ▪ One lecture in Paeds, but I never got to see or do ▪ No previous learning ▪ Practice on model in 3rd year Have not done any, so most of this in non-existent ▪ We just had a dummy vagina in MED3 to learn speculum and bimanual exam. A total of 3 h max	Positive Experience with suggestions for minor change ▪ I enjoyed it! ▪ Very valuable. ▪ It is a good addition and should be continued ▪ Fantastic module ▪ Scary, but helpful ▪ Specific advice around technique adjustment was really useful! ▪ Invaluable learning opportunity. Very helpful tutors and empathetic actors. ▪ This was easily the best part of the Module. Very valuable. The pre-exam video for males is poor and should be replaced. ▪ Would be good to have a range of ages and subjects and more exposure More instruction/demonstration required prior to having to do examination ▪ It was good although I would’ve liked to be able to see the genital exam performed and explained before having to do it ourselves. ▪ A bit more formal instruction before exam would have helped. Quality of experience dependent on tutor ▪ Dr X was absolutely fantastic as a tutor ▪ Dr Y was rude to both me and the teaching associate. I felt this was inappropriate and unconstructive.

## Discussion

### Main findings

This cohort of senior medical students reported greatly improved skill, confidence and comfort levels for both male and female genital examination following the teaching programme. Skill, confidence and comfort regarding male examinations were rated particularly low on the pre-teaching programme self- assessment, but post-programme was rated at similar improved levels to the female examination. Student gender analysis revealed that male students rated themselves slightly higher than female students in both pre and post programme scores. Interestingly males felt less skilled examining males than they did females in the pre-programme scores, although this was not statistically significant.

The teaching programme also had a positive impact on how students rated factors such as the adequacy of their education/instruction, preparedness for examination, opportunities for observation of and participation in examination, and the level of supervision and feedback from instructors.

Themes from the free text comments fields indicate that prior to the new teaching programme being introduced students had limited opportunistic or no opportunities for genital examinations, particularly male examinations. Comments about the teaching programme centred around what a positive experience it was. However, the quality of the individual tutor had a big impact on the experience and there was a perceived need by some students for more instruction/demonstration prior to completing their first intimate examination.

### Strengths and limitations

The strengths of the programme were the integration of both male and female genital examination education and the use of a variety of teaching methods. Many medical schools offer opportunities for female genital examination but the use of simulated patients for the teaching of male genital examination is less common. An integrated programme of learning including both male and female genital examination in a simulated educational setting has been rarely reported [17, 18].

In the study, the overall response rate for matched pre and post results was favourably comparable with similar studies [16]. However, not all of the students’ pre and post questionnaires were able to be matched (26%), and it is not known whether this is due to students not wishing to participate or an error on the student’s part when completing the questionnaires. However 74% of eligible students did fully participate. While it was possible to undertake a simple gender analysis, it was not possible to further analyse data by ethnic group due to small numbers. Although this study was limited to student self-assessment, perceived increase in confidence and competence is an important first step in any skills learning process [19, 20] and is particularly important when learning genital examination skills in both genders. This was a point in time survey and another question to be addressed is how long the effects lasted.

### Implications

Previous systematic reviews have identified improved short term outcomes for student learning with the addition of a standardised patient [8, 13], however evidence of longer term impact is still limited [8]. Further research assessing doctors in the first year of practice may be useful.

Only two other studies have included teaching programmes with both male and female genital examination together in the same programme (pelvic, rectal, breast and testicular), [17, 18] with most studies focusing on either male or female anatomy separately. The undertaking of both male and female examinations as part of an integrated programme also has important connotations for both male and female medical students. All examine a TA of the opposite, and the same, gender as themselves. This helps to minimise any perception that either gender is more important, and as an aside, may also on occasion (especially for younger students) act as a valuable learning experience about aspects of their own anatomy and sexual health.

The findings from the evaluation have enabled ongoing development of the teaching programme in subsequent years. Clinical tutors have now been given more directed guidance on their role in the session. One unanswered question is how much formal teaching is required or if the guided learning is enough to enable students to have the confidence to enhance their skills as clinical situations present in subsequent training or practice. Formal feedback from the TAs and clinical tutors may also be useful in programme development. In some medical teaching settings the teaching is undertaken by the TA without a tutor [10, 11, 21]. This may be worth exploring.

## Conclusions

With the student demographic profile becoming increasingly diverse, and better reflecting the general New Zealand population (including ethnic mix, cultural norms and gender balance) it is important to try new approaches to genital examination training which provide a safe, supported and relaxed environment for approaching this sensitive area of learning and appropriate focus on both male and female anatomy. This teaching programme, utilising teaching associates in a simulated clinical environment, was successful in providing senior medical students with increased skill, confidence and comfort in performing genital examinations for both males and females as part of their clinically-based rotations. Suggestions for improvement included giving more detailed instruction to clinical supervisors about their role in the examination process and having a demonstration of the exam on male and female patients prior to students undertaking their first examination. Future analysis and work will include looking at the more longitudinal benefits of the program for the students and also seeking more formal feedback from the tutors and TA’s.

## Additional files

Additional file 1: Evaluation survey of student experience with genital examination (Pre). This is the questionnaire administered to the students prior to the teaching programme. (DOCX 21 kb)
Additional file 2: Evaluation survey of student experience with genital examination (Post). This is the questionnaire administered to the students after the teaching programme. (DOC 58 kb)

## Abbreviations

GTAs: Gynaecological teaching associates
O&G: Obstetrics and Gynaecology
PHC&GP: Primary Health Care and General Practice
TAs: Teaching associates
WSHS: Wellington Sexual Health Service
