# Supplementary material for: Genital examination training: assessing the effectiveness of an integrated female and male teaching programme
Source: BMC Med Educ. 2016 Nov 22;16:299. doi: 10.1186/s12909-016-0822-y (PMC5120523; doi:10.1186/s12909-016-0822-y)
Supplement: Additional file 2: — Evaluation survey of student experience with genital examination (Post). This is the questionnaire administered to the students after the teaching programme. (DOC 58 kb) [file 12909_2016_822_MOESM2_ESM.doc]

**Post survey of student experience with genital examination.**

**All questions relate to your genital examination learning and experience having completed this Year 5 GP module.**

| **Please include the last six (6) digits of your mobile phone number. This will be used to anonymously code your survey:** | | | | | | | | |
| --- | --- | --- | --- | --- | --- | --- | --- | --- |
|  | | | | | | | | |
| **Please indicate your gender :** | | | | | | | | |
|  | Female | |  | Male | | | | |
| **The following questions relate to your learning experience of genital examination as part of your year 5 GP Module. Please select the one response with a tick (√) that is most relevant to you for each question:** | | | | | | | | |
| **How would you best rate:** | | | **Non-existent** | | **Poor** | **Adequate** | **Good** | **Excellent** |
| 1. The adequacy of education to support your learning about genital examination? | | |  | |  |  |  |  |
| 2. The adequacy of educational materials (i.e. books, videos, access to internet and so forth) to support your learning about genital examination? | | |  | |  |  |  |  |
| 3. The quality of dedicated instruction you received in relation to the genital examinations? | | |  | |  |  |  |  |
| 4. The level of preparedness to complete genital examinations? | | |  | |  |  |  |  |
| 5. The opportunities to observe genital examinations? | | |  | |  |  |  |  |
| 6. The genital examination opportunities offered as part of this module? | | |  | |  |  |  |  |
| 7. The supervision offered by clinicians with the genital examinations as part of this module? | | |  | |  |  |  |  |
| 8. The level of feedback you have received following your genital examinations? | | |  | |  |  |  |  |
| 9. Your skill level with genital examinations? | | Male |  | |  |  |  |  |
|  |  | Female |  | |  |  |  |  |
| 10. Your confidence to complete genital examinations? | | Male |  | |  |  |  |  |
|  |  | Female |  | |  |  |  |  |
| 11. Your comfort with completing genital examinations? | | Male |  | |  |  |  |  |
|  |  | Female |  | |  |  |  |  |
| **Please include any additional comments relating to your learning and experience with genital examinations in this Year 5 GP module:**  NB Please use the back of this page if you require further space for comments. | | | | | | | | |
